# An efficient and compromise-resilient image encryption scheme for resource-constrained environments

**DOI:** 10.1371/journal.pone.0297534

**Published:** 2024-04-18

**Authors:** Abdul Nasir Khan, Abid Mehmood, Muhammad Nasir Mumtaz Bhutta, Iftikhar Ahmed Khan, Atta ur Rehman Khan

**Affiliations:** 1 COMSATS University Islamabad, Abbottabad Campus, Khyber Pakhtunkhwa, Pakistan; 2 Department of Computer Science and Information Technology, College of Engineering, Abu Dhabi University, Abu Dhabi, UAE; 3 College of Engineering and IT, Ajman University, Ajman, UAE; University of Sindh, PAKISTAN

## Abstract

The secret keys produced by current image cryptosystems, which rely on chaotic sequences, exhibit a direct correlation with the size of the image. As the image dimensions expand, the generation of extensive chaotic sequences in the encryption and decryption procedures becomes more computationally intensive. Secondly, a common problem in existing image encryption schemes is the compromise between privacy and efficiency. Some existing lightweight schemes reveal patterns in encrypted images, while others impose heavy computational burdens during encryption/decryption due to the need for large chaotic sequences. In this study, we introduce a lightweight image encryption scheme that involves partitioning the image into uniformly sized tiles and generating a chaotic sequence accordingly. This approach diminishes the necessity to create extensive chaotic sequences equal to the tile size, which is significantly smaller than the original image. As a result, it alleviates the processing burden associated with generating sequences equivalent to the original image size. The results confirm that our proposed scheme is lightweight and secure compared to the latest state-of-the-art image encryption schemes. Additionally, sensitivity analysis demonstrates that the proposed image encryption technique, with a UACI value of 33.48 and NPRC value of 99.96, affirms its resistance to differential attacks.

## 1. Introduction

**Fig 1:**
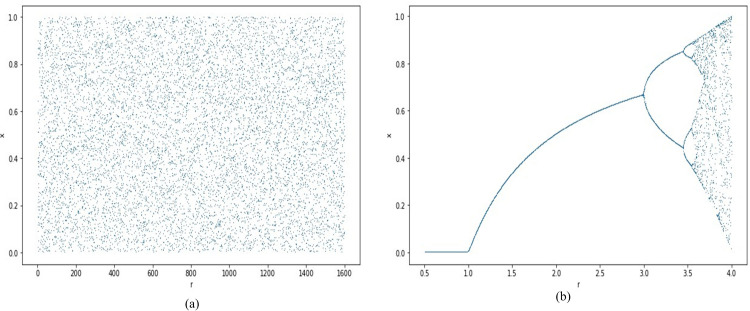
Bifurcation diagrams of (a) JSMP Map and (b) Logistic Map.

The properties of chaotic maps, such as pseudo-randomness, deterministic, non-linearity, and sensitivity to initial conditions, attracted the research to use in image cryptography. There are two types of chaotic maps: (a) discrete-time iterative map system and (b) continuous time differential equation map system. The discrete-time iterative map system represents the evaluation of the system at discrete intervals of time. These types of maps are usually represented with the iterative equation. Examples of discrete maps are logistic [1] and sine maps [2]. The continuous time differential equation map is used to represent the evaluation of the system at a continuous function of time. These types of maps are described using ordinary differential equations. Lorenz system [3] and Chen system [4] are examples of differential equation maps. Both types of chaotic systems can be used in image cryptography. Discrete-time iterative map systems are usually used to generate a pseudorandom sequence, which may be used as a key for enciphering the image or shuffling the image pixels based on the generated sequences. Similarly, continuous time differential equation maps are used to produce a continuous chaotic signal, which is transformed into discrete data for the encryption of the image. In this research study, we focus on discrete-time iterative map systems because these systems require less processing and are easier to implement than continuous time differential equation maps. There are many single-dimension and multi-dimension discrete-time iterative maps proposed. Single-dimensional discrete-time iterative maps generate only a single chaotic sequence, while multi-dimensional discrete-time iterative maps generate multiple chaotic sequences simultaneously. The low-dimensional discrete-time iterative map system usually requires less processing power than the high-dimensional discrete-time iterative map system. Hence, low-dimensional systems are typically preferred in light-weight cryptosystems. The chaotic regime is another critical factor in selecting a low-dimensional discrete-time iterative map system. If the chaotic regime of a system is high, then the key space is large, and the system protects against brute force attacks. Fig 1 shows the chaotic regime of logistic and JSMP maps [5].

This diagram shows the mapping of 1000 values generated using the logistic and JSMP maps. The figure shows that both maps behave chaotically, but the logistic map’s chaotic regime is much shorter than the JSMP map. The map with the shorter chaotic regime is suspected to a brute force attack.

The applications of chaotic maps in image cryptography are diverse, spanning from Pseudo Random Number Generation (PRNG), key generation, and image transformation to confusion and diffusion. In recent research, chaotic fractional-order differential systems like the Chen system, Chua system, and memristor system have been used to generate random numbers. These systems produce multiple numerical sequences for pseudo-randomness, which are subsequently consolidated into a single-bit stream through processes involving flattening and boolean checking [6]. Additionally, the incorporation of numerous control variables in chaotic fractional-order differential systems expands the key space, adding complexity and making brute-force attacks challenging. In numerous image encryption research, chaotic maps are instrumental in generating key sequences, subsequently adjusting these sequences to the required range through module reduction. The selection of a chaotic map for key generation hinges on two crucial factors: the chaotic regime and initial parameters. A sizable chaotic regime renders brute-force attacks impractical, and the inclusion of multiple initial parameters enhances the scheme’s security since these parameters serve as secret keys. Nevertheless, securely distributing these initial parameters presents a challenging task. During the image transformation phase, a cipher image is generated by scrambling the key stream, produced through a chaotic map, and the original image. This scrambling process can take place at the bit level using operations such as xor or xnor, similar to a stream cipher. It can also operate on two bits using xor, xnor, or binary addition/subtraction operations, as seen in DNA encoding [7]. Alternatively, the process may extend to n bits, involving xor, xnor, or binary addition/subtraction, along with bit rotation operations. The fundamental requirement for the core encryption operation is reversibility. However, employing multiple operations in the encryption algorithm enhances the diffusion property, contributing to a more effective transformation. Confusion is achieved through the incorporation of substitution boxes [8]. In most of the existing image encryption approaches, a 16 × 16 S-Box is generated using a chaotic map. Given that the pixel intensity of an image falls within the range of 0 to 255, and the core encryption operation operates on eight bits (where n equals eight), the S-Box size is maintained at 16 × 16. Alternatively, some recent research studies adopt the use of multiple parallel S-Boxes with a size denoted as m for substitution operations. The majority of current image encryption schemes leverage chaotic sequences to execute nearly all the operations mentioned above, enhancing the reliability and security of the scheme [9, 10].

Many sensitive applications send image data on the Internet, such as healthcare systems, Internet of Battlefields Things (IoBTs) [11], Internet of Multimedia Things (IoMTs) [12], Industrial Automation [13], Security Surveillance [14], and many more. The privacy of image data is critical in such applications because disclosure of the information may result in financial or territorial loss. There are many applications where the privacy of image data is important, but traditional cryptographic schemes are unsuitable due to the resource-constrained environment. Hence, A novel, lightweight, and secure image encryption scheme is needed. In this research study, we

➢ examine the shortcomings of existing lightweight image encryption approaches.
➢ propose a secure and lightweight image encryption method utilizing a JSMP map,
➢ conduct a comparative evaluation with proposed image encryption schemes using metrics like encryption/decryption times, histogram analysis, correlation analysis, entropy analysis, sensitivity analysis, and time complexity analysis.
➢ conduct diverse analyses, including key space analysis and security assessment, to demonstrate the efficacy of the proposed image encryption technique in thwarting various attacks.
➢ validate the proposed image encryption technique by subjecting it to NIST 800–22 test suit.

The rest of the paper is organized as follows: section 2 discusses the related work on existing image encryption schemes, section 3 contains the proposed image encryption schemes, section 4 compares the proposed image encryption scheme with existing schemes based on performance parameters, and Section 5 concludes the research.

## 2. Related work

In this research study, our focus is to propose a lightweight image encryption scheme using chaos cryptography. Many research articles have been published in reputed journals on image encryption using chaos cryptography, such as [15–17]. The existing image-based encryption techniques can be divided into symmetric chaos-based image encryption schemes [18–23], asymmetric chaos-based image encryption schemes [24–27], and hybrid chaos-based image encryption schemes [28, 29]. This research focuses on symmetric chaos-based image encryption, subdivided into spatial domain [30] and frequency domain [31–33]. Spatial domain image encryption schemes directly work on pixels to get the cipher image. In contrast, frequency-domain image encryption schemes transform the plain image into the frequency domain using mathematical transformation before encrypting the image. The spatial domain image encryption techniques are fast and do not distort the decrypted image. Therefore, we have selected four recently published spatial domain image encryption schemes [34–37], discussed below.

**Fig 2:**
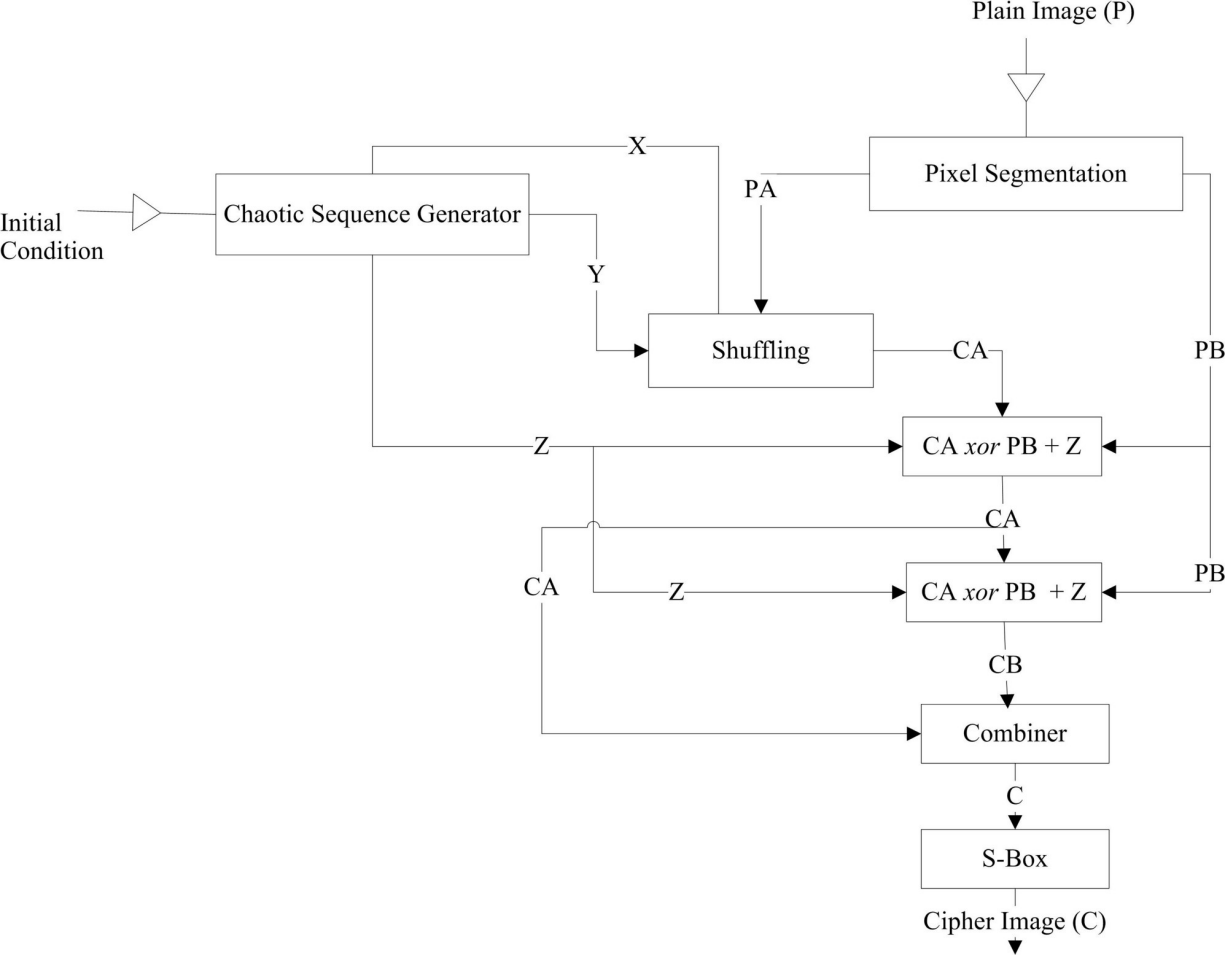
Flow of image encryption scheme of [37].

In [37], authors proposed a new robust chaotic map by combining quadratic parabolic and piecewise functions to construct a non-linear system and strong S-box. Subsequently, the authors use the newly proposed map and S-Box to propose a new image encryption scheme. A new proposed hybrid approach generates three chaotic sequences: *X*, *Y*, and *Z*. *X* is equal to *M*, *Y* is equal to *N*, and *Z* is equal to *M* × *N*, where *M* and *N* represent the rows and columns of an original image, respectively. In this proposed technique, the hash value of the original image is transformed into a value used as an initial condition to generate chaotic sequences. As the chaotic sequences are very sensitive to the initial condition, a minor change in the original image generates an entirely different chaotic sequence, *X*, *Y*, and *Z*. The encryption process starts by dividing the plain image *P* into two sub-images, *PA* and *PB*, where *PA* contains the higher four bits of each pixel of *P* and *PB* contains the lower four bits of each pixel of *P*. The value of *PA* is shuffled based on *X* and *Y*. The shuffled PA is further encrypted by performing the exclusive-or operation on *PA*, shuffled *PA*, and *Z* to get the *CA*. *CB* is generated by performing the exclusive-or operation on *CA*, *PB*, and *Z*. Then, the encrypted form of sub-image *CA* and *CB* are combined to get the temporary encrypted image *C*. To achieve confusion and diffusion, each pixel of the temporary encrypted image is shuffled based on S-Box and then added with previously shuffled pixels and *Z* to get the final encrypted image. The flow of the image encryption scheme is shown in Fig 2.

The key sequences generated in this scheme depend on the original image’s size. If the size of the plain image increases, it takes more time to generate the sequences. Moreover, pixel segmentation, scrambling, pixel splicing, confusion, and diffusion processes during encryption make this scheme computationally intensive.

**Fig 3:**
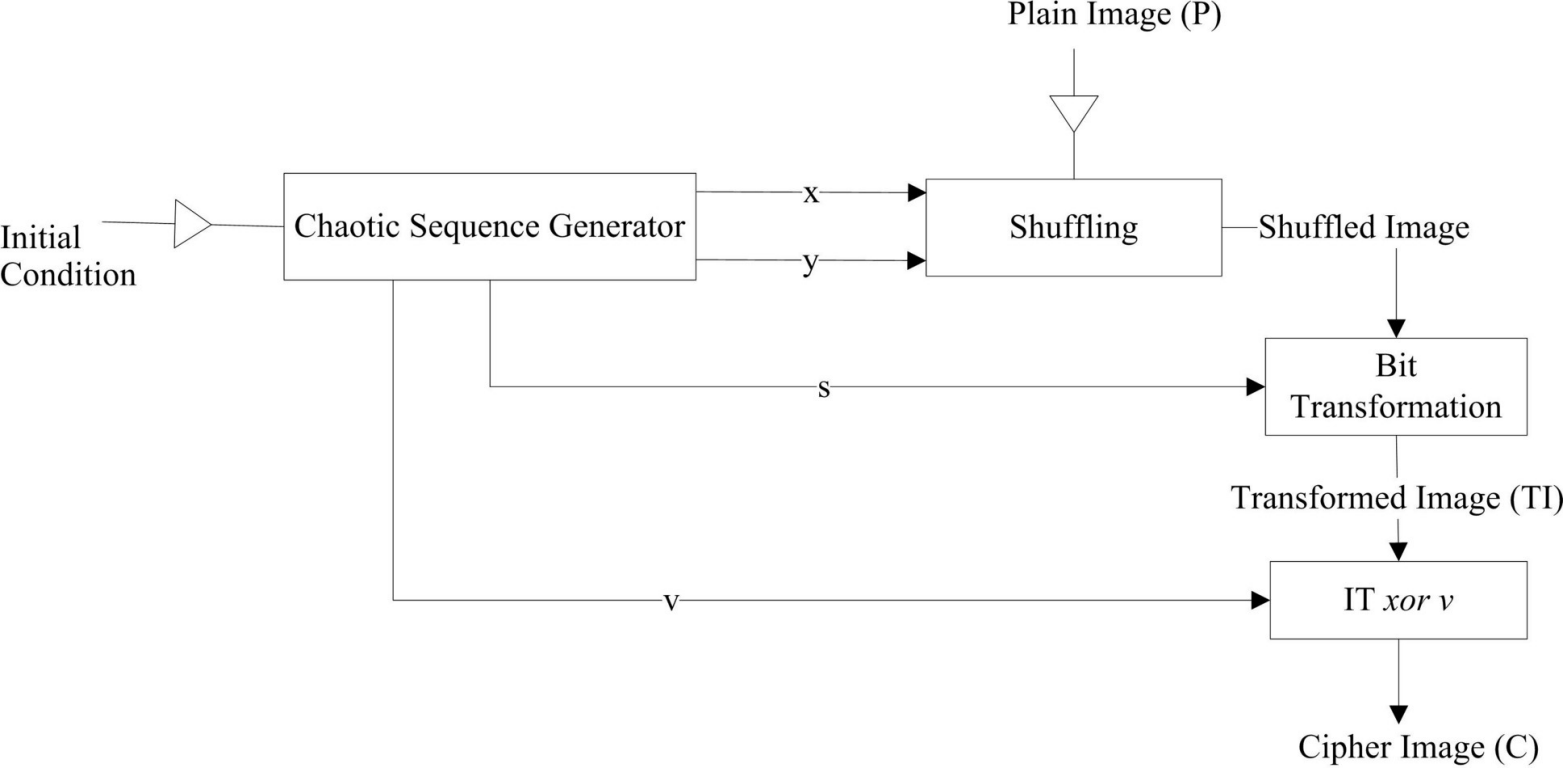
Flow of image encryption scheme of [35].

Authors in [35] proposed a lightweight image encryption scheme and 1D quadratic chaotic map to generate key sequences. Two main modules are involved in the proposed scheme: shuffling and substitution. Two sequences, *x* and *y*, of size *M × 10*^*3*^ and *N×10*^*3*^ are generated for shuffling the image. The *x* sequence is used to shuffle the rows of the original image, and the *y* sequence is used to shuffle the columns of the plain image. After shuffling the image, two more sequences, *s* and *v*, of size *8 × M × N* and *M × N* are generated for the substitution module. The first step of the substitution module is bit transformation, which uses sequence *s*. Secondly, the bit transformed image is exclusive-or with v to get the final cipher image. The flow of the proposed scheme is presented in Fig 3.

**Fig 4:**
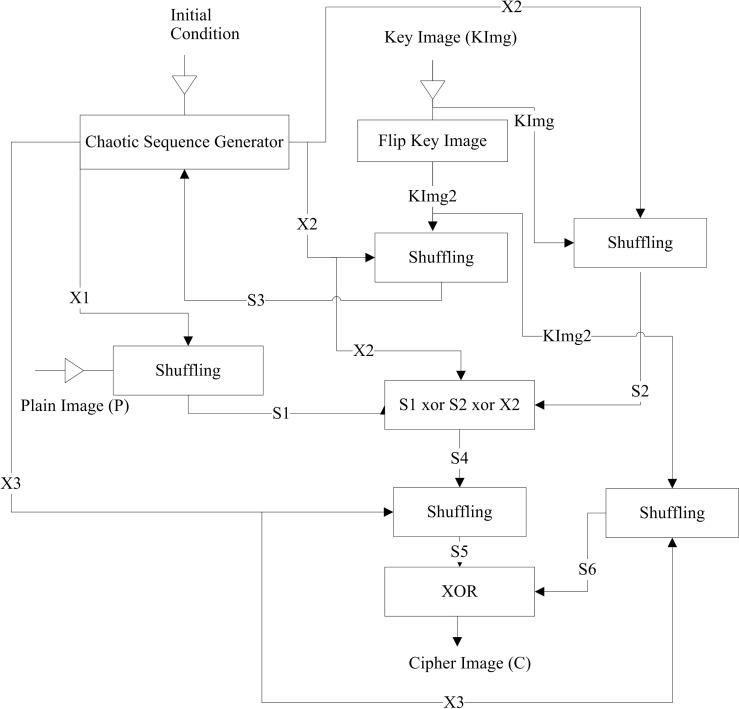
Flow of image encryption scheme of [34].

In the [34] proposed scheme, three chaotic sequences are generated: *X1*, *X2*, and *X3* of size *M+N*. The sequence *X1* is generated using an Improved Logistic Map (ILM), *X2* is generated using the Logistic Mayan System (LOMAS), and *X3* is generated using an Improved Sinusoidal Map (ISM)—the key used in this research study in an image. The key and original image are input to the encryption along with the initial conditions for the chaotic systems. The initial condition parameters are generated by taking the hash value of the original image. In the first phase of image encryption, two streams, *S1* and *S2*, are generated by permuting original and key images based on *X1* and *X2*, respectively. The *S3* stream is generated by rotating the key image 180 and then permuting it based on *X2*. Then *S4* is generated by taking the exclusive-or of *S1*, *S2*, and *X2*. *S4* is permuted based on *X3* to get the value of *S5*, and a 180-degree rotated key image is permuted based on *X3* to get the stream *S6*. Finally, *S5* and *S6* streams are exclusive-or to get the final encrypted image. The flow of the scheme is shown in Fig 4.

**Fig 5:**
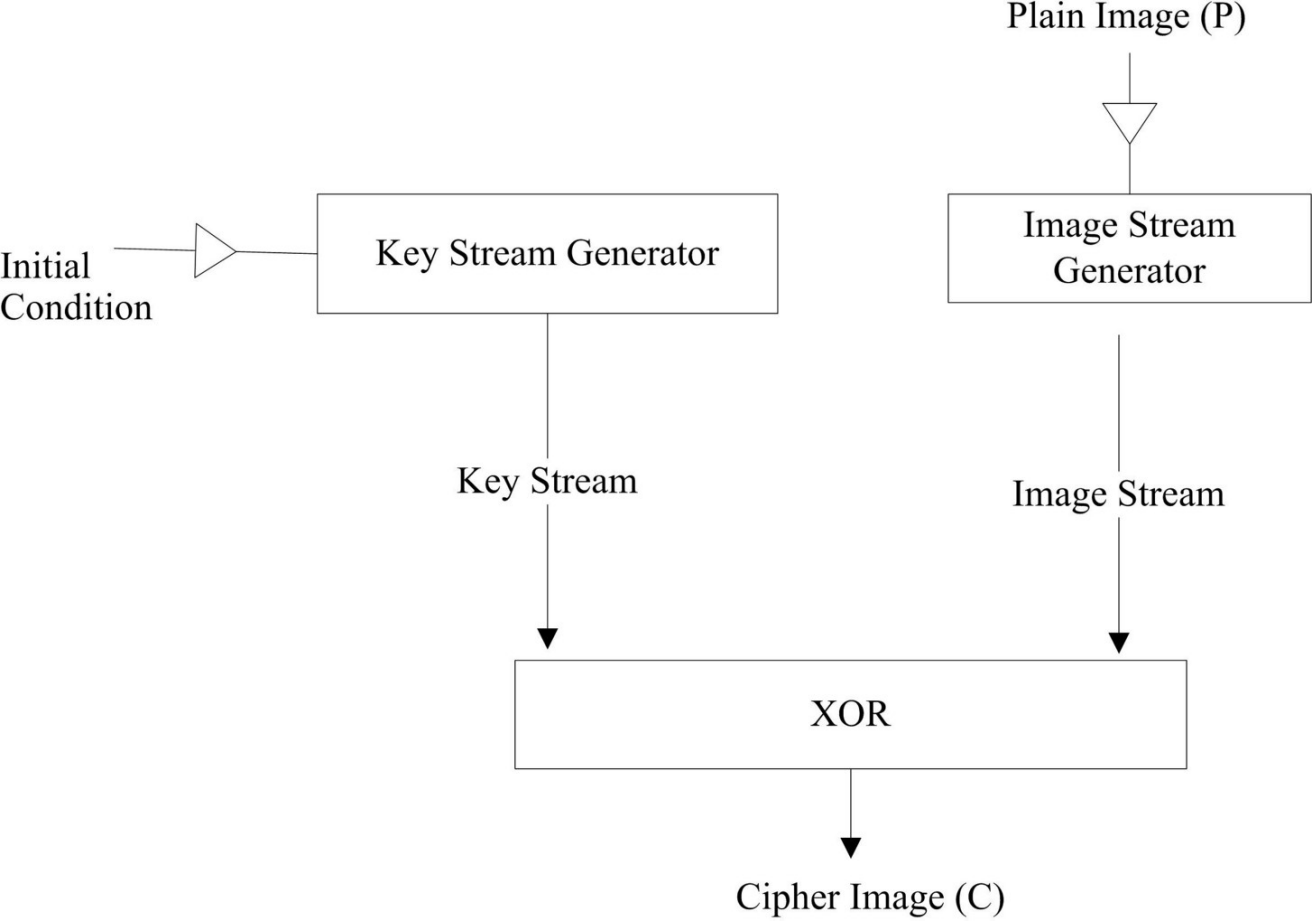
Flow of image encryption scheme of [36].

The authors of [36] proposed a new chaotic map-based random number generator module for a keystream generator. The image is transformed as a byte stream. The image stream and keystream are exclusive-or to get the final stream. The final stream is converted into a cipher image. The flow of the scheme is shown in Fig 5.

**Fig 6:**
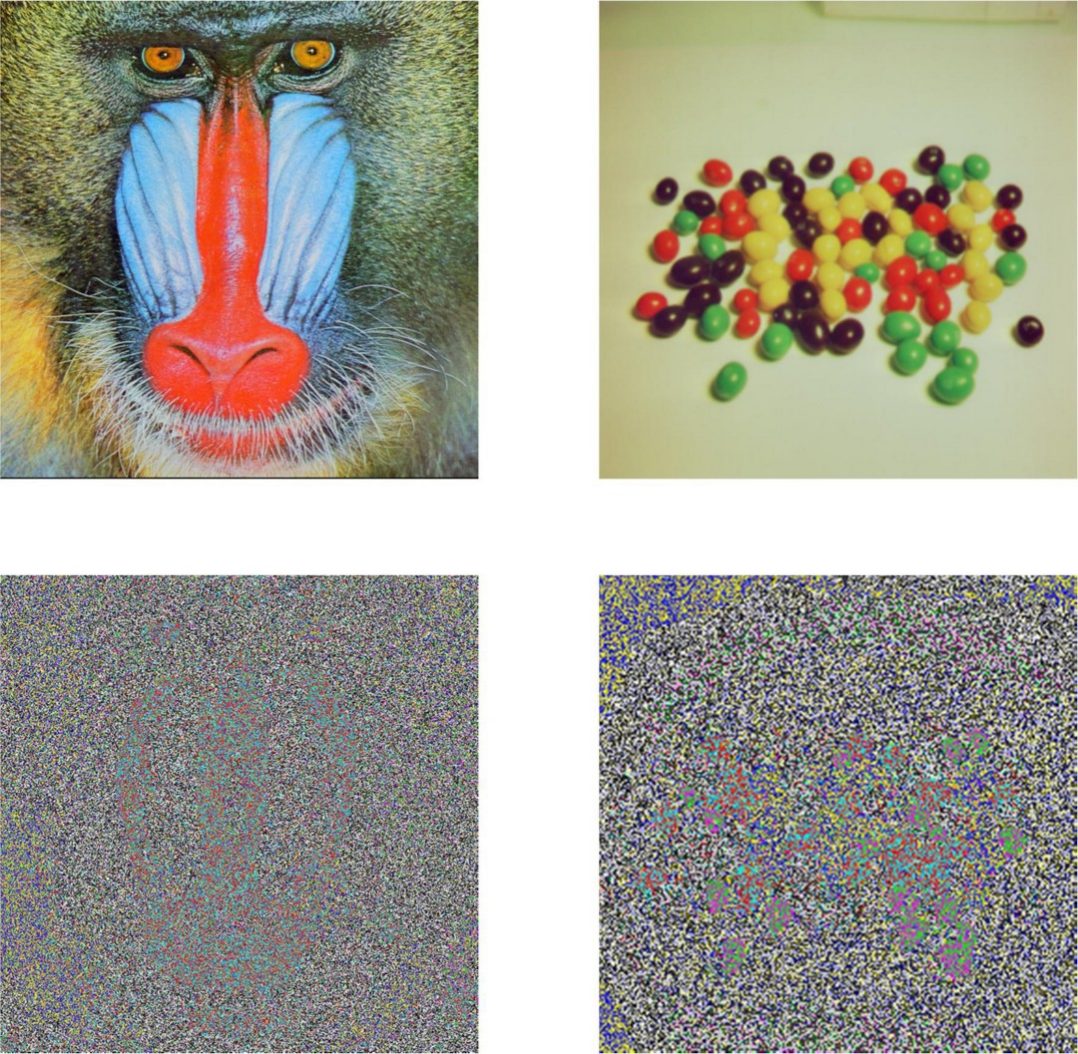
Encrypted images using [36].

The proposed technique is lightweight in terms of processing; however, it reveals the pattern in encrypted image, as shown in Fig 6.

The chaotic sequences used in existing schemes are presented in Table 1.

**Table 1:** Chaotic sequences of existing schemes.

	Image of Size M × N			
	Sequence 1	Sequence 2	Sequence 3	Sequence 4
**[37]**	*X* of size *M*	*Y* of size *N*	*Z* of size *M* × *N*	-
**[35]**	*x* of size *M × 10*^*3*^	*y* of size *N×10*^*3*^	*s* of size *8 × M × N*	*v* of size *M × N*
**[34]**	*X1* of size M+N	*X2* of size M+N	*X3* of size *M+N*	—
**[36]**	Key Stream of Size *M × N*	-		

The existing image encryption schemes compromise privacy by disclosing the pattern in encrypted images or imposing the processing burden during encryption/decryption by generating large chaotic sequences, as shown in Table 1. In most schemes, the size of a chaotic sequence is equal to the number of pixels of a plain image. Even in some existing schemes, the recommended chaotic sequence size is more than the number of pixels of the plain image. Hence, there is a direct relationship between the size of the chaotic sequence and the number of pixels of the plain image. Such schemes are not suitable for resource-constrained environments, such as IoTs. Hence, resource-constrained computing environments need lightweight, secure image encryption schemes. In this research study, we proposed a novel lightweight and compromise-resilient image encryption scheme. The proposed scheme is compared with existing image encryption based on processing time, entropy, Mean Square Error (MSE), correlation analysis, sensitivity analysis, and Peak signal-to-noise ratio.

## 3. The proposed lightweight and compromise resilient image encryption scheme

**Fig 7:**
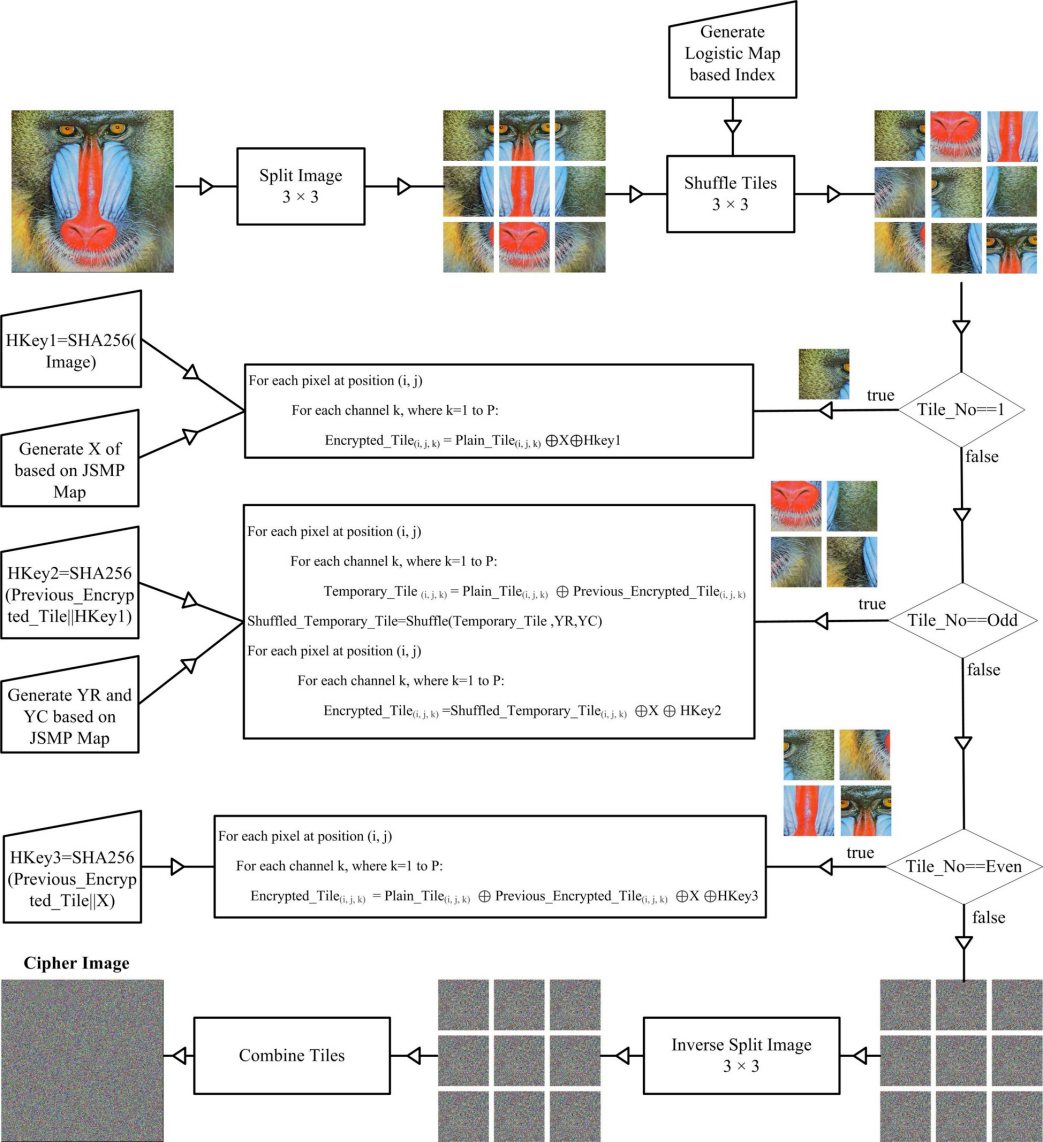
Encryption process for the proposed image encryption scheme.

The flowchart depicting the proposed image encryption scheme is presented in Fig 7. The proposed technique comprises eight distinct modules, specifically: (a) Image Splitter, (b) Key Generator, (c) Index Generator, (d) Random Number Generator, (e) Tiles Shuffler, (f) Pixels Shuffler, (g) Encryption, and (h) Decryption modules.

Initially, the plain image is segmented into tiles of uniform size with dimensions represented by *M*_*t*_ × *N*_*t*_, where *M*_*t*_ and *N*_*t*_ denote the number of rows and columns in each tile. The tile height and tile width are computed by dividing the image height with *M*_*t*_ and image width with *N*_*t*_, respectively. Subsequently, key sequences are generated utilizing the JSMP chaotic map. Mainly, three sequences are employed: X, YC, and YR, using JSMP chaotic map. The size of sequence *X* matches that of the tiles, *M*_*t*_ × *N*_*t*_, and it is used for encrypting the tiles through the exclusive-or operation. The process for generating sequence *X* is detailed in algorithm 1. Many existing one-dimensional chaotic map generators fail to provide an extensive chaotic regime, resulting in limited key space vulnerability to brute force attacks. In the proposed scheme, keys are generated using the one-dimensional JSMP [5] chaotic map, which offers a broader chaotic regime and an enhanced key space. The key generation algorithm utilizing the JSMP chaotic map is illustrated below:

**Algorithm 1** *keyGenerator(x*_*0*_, *r*, *S)*: Generating Key based on JSMP Chaotic Sequence
**Input:** initial value of *x*_*0*_, control parameter *r*, and key size *S*
**Output:** Chaotic Sequence based key *key* ← *[key*_*1*_, *key*_*2*_, *key*_*3*_, *key*_*4*,*……*.,_, *key*_*s*_*]*
    for i ← 1 to S
         $x_{i}\leftarrow \left(r^{2}\times \left(x_{n-1}^{2}-5\right)\times \left(1-\text{r}\left(x_{n-1}^{2}-5\right)\right)mod1\right.$
      $key_{i}\leftarrow \left(x_{i}\times 10^{12}\right)mod256$
    endfor
return *key*

The generated value of *x*_*i*_ falls within the range of 0 to 1. This generated *x*_*i*_ value is converted into an integer and reduced by applying a modulo operation. Each key-value thus obtained falls within the range of 0 to 255, mirroring the range of image pixels. *YC* and *YR* represent index sequences sized at *M*_*t*_ and *N*_*t*_, respectively. These sequences are generated to reorganize the pixel values within individual tiles. The process for generating *YC* and *YR* is elucidated in Algorithm 2. The index values within these sequences range from *0* to *S-1*. The purpose of the chaotic sequence is to reorder the index values based on the generated chaotic sequence, thereby introducing a shuffling effect.

**Algorithm 2** *indexGenerator(x*_*0*_, *r*, *S*): Creating an index for image shuffling using a JSMP Chaotic Sequence
**Input:** initial value of *x*_*0*_, control parameter *r*, and key size *S*
**Output:** Chaotic Sequence based key *index = [index*_*0*,_ *index*_*1*_, *index*_*2*_, *index*_*3*_, *index*_*4*,*……*.,_, *index*_*s-1*_*]*
    for i ← 0 to S-1
      $x_{i}\leftarrow \left(r^{2}\times \left(x_{i-1}^{2}-5\right)\times \left(1-\text{r}\left(x_{i-1}^{2}-5\right)\right)mod1\right.$
      $\;key_{i}\leftarrow \left(\left[x_{i}\times 10^{12}\right]\right)mod256$
      *index*_*i*_←*i*
    endfor
    for *i* ← 0 to *S-1*
      for *i* ← 0 to *S-1*
            if *key*_*i*_ > *key*_*j*_, then
                swap(*key*_i,_ *key*_*j*_*)*
                swap(*index*_i,_ *index*_*j*_)
            endif
      endfor
    endfor
return *index*

The proposed scheme employs three discrete logistic map-based random number generators as logistic maps are suspected of brute force attacks due to their small chaotic regime. Therefore, it generates random numbers only to select a byte from a 256-hash value. Hence, if the attacker finds the key to generating a logistic map, he cannot decrypt the image. Each method produces a distinct sequence of numbers, even when provided with identical initial parameters. These algorithms generate random numbers ranging from 0 to 31, which are utilized to shuffle the produced hash values in the encryption process. Further information about these random number generators is provided in Algorithms 3, 4, and 5.

**Algorithm 3** *GenRanNum1(x*, *r*,*)*: Generating Random Number using Logistic Chaotic Sequence
**Input:** initial value of *x*, control parameter *r*
**Output:** Random number *rand* and updated value of *x*
    *x*←(*r* × x ×(1 − x))
    *rand*←([*x*] × 2^12^)*mod*32
return *rand*, *x*

The first random number generator produces a value for ’x’ within the range of 0 to 1, which is subsequently converted into an integer and then subjected to modulo operations. This algorithm derives the random value from the integral part while ignoring the fractional component. The value of *x* is also returned with *rand* to use as an input for the next call to this function.

**Algorithm 4** *GenRanNum2(x*, *r*,*)*: Generating Random Numbers through Logistic Chaotic Sequences
**Input:** initial value of *x*, control parameter *r*
**Output:** Random number *rand* and updated value of *x*
    *x*←(*r* × x × (1 − x))
    *rand*←(*x* × 1000000 –[*x* × 1000000])*mod*32
return *rand*, *x*

Algorithm 4 generates the random value based on the fractional part, and the integral part is ignored.

**Algorithm 5** *GenRanNum3(x*, *r*,*)*: Generating Random Number using Logistic Chaotic Sequence
**Input:** initial value of *x*, control parameter *r*
**Output:** Random number *rand* and updated value of *x*
    *x*←(*r* × x × (1 − x))
    *rand*←(([*x*] × 2^12^) + (*x* × 1000000 − [*x* × 1000000]))*mod*32
return *rand*, *x*

Similarly, Algorithm 5 generates the random value based on the fractional and the integral parts.

The image is partitioned into uniform-sized tiles, which are reorganized using the method elucidated in Algorithm 6. This algorithm generates a logistic chaotic map to produce a random sequence of indexes spanning from 0 to D-1, with ’D’ representing the total number of tiles in the original image. It’s important to note that this algorithm is specifically designed for cases where ’D’ is an odd value.

**Algorithm 6** *ShuffleTiles(tiles*, *D*, *x*, *r)*: Shuffle the tiles of original image based on index
**Input:** initial value of *x*, control parameter *r*, *tiles of original image*, *D is count of tiles*
**Output:** shuffled tiles *s_tiles*
*index* ← *indexGenerator(x*, *r*, *D*):
    for *i* ← *0* to *D-1*
      $s_{-}tiles_{i}\leftarrow \;tiles_{index_{i}}$
    endfor
return *s_tiles*

The procedure for reversing the tile shuffling is detailed in Algorithm 7.

**Algorithm 7** *InverseShuffleTiles(stiles*, *D*, *x*, *r)*: Arrange the tiles of an image in its original form
**Input:** initial value of *x*, control parameter *r*, *stiles shuffled tiles of image*, *D is count of tiles*
**Output:** tiles in its original position
*index = indexGenerator(x*, *r*, *D*):
    for *i* ← *0* to *D-1*
      $\;tiles_{index_{i}}\leftarrow s_{-}tiles_{i}$
    endfor
return *tiles*

Specific tile pixels are also subjected to shuffling to introduce confusion and diffusion into the encrypted image. Algorithm 8 illustrates the procedure for shuffling the pixels within a tile, wherein the shuffling is determined by the chaotic sequences *YC* and *YR*.

**Algorithm 8** *ShuffleTilePixels(tile*, *YC*,*YR*, *R*_*t*_, *C*_*t*_*)*: Shuffle the pixels of a tile
**Input:** tile of an image is represented with *tile*, *YC* and *YR* are generated using *IndexGenerator()* for shuffling of pixels inside tiles, *and R*_*t*_ *and C*_*t*_ *represents the rows and cols of tile respectively*
**Output:** shuffled tiles *s_tile*
    for *i* ← 0 to *R*_*t*_ -1
      for *j* ← 0 to *C*_*t*_ -1
           *s*_*tile*_*i*,*j*_←*tile_YR[i],YC[j]_*
      endfor
    endfor
return *s_tile*

The inverse process of pixel shuffling is shown in Algorithm 9.

**Algorithm 9** *InverseShuffleTilePixels(s_tile*, *YC*,*YR*, *R*_*t*_, *C*_*t*_*)*: Arrange the pixels of a tile in its original form
**Input:** shuffled tile of an image is represented with s_*tile*, *YC* and *YR* are generated using *IndexGenerator()* for shuffling of pixels inside tiles, and *R*_*t*_ and *C*_*t*_ represents the rows and cols of tile respectively
**Output:** tile in its original form *tile*
for *i* ← 0 to *R*_*t*_ -1
    for *j* ← 0 to *C*_*t*_ -1
      *tile_YR[i],YC[j]_*←*s_tile*_*i*,*j*_
    endfor
endfor
return *tile*

The encryption and decryption algorithm generates a hash value using SHA256, resulting in a hexadecimal representation. This hexadecimal value is then converted into an integer array comprising 32 elements, which play a crucial role in the encryption process. The conversion process for the 256-bit hexadecimal hash value is detailed in Algorithm 10.

**Algorithm 10** *GetHashValue(Image*, *data)*: generates the hash value on Image and data and convert it into an integer array of 32 elements contains the value form 0 to 255
**Input:** *Image and data*
**Output:** *hkey* is hash value of image and data in integer form
*h* = SHA256(*Image+Data*)
    for *i* ← 0 to *len(h/2)*
         *val1← convertHextoInt(h*_i_*)*
         *val2← convertHextoInt(h*_i+1_*)*
         *hkey*_*i*_←(*val*1×16) + *val*2
    endfor
return *hkey*

The encryption algorithm takes the original image as input and transforms it into a cipher image. The process of image encryption is illustrated in Algorithm 11. To initiate the encryption process, the plain image is initially divided into *D = V* × *V* tiles, where *V* is an odd integer. These tiled images are then subjected to shuffling, as explained earlier. Three distinct mechanisms are incorporated to introduce randomness and enhance protection against differential attacks. Before encrypting the first tile, hkey1 is generated by applying SHA256 to the plain image. The resulting SHA256 hexadecimal value is converted into an integer array of 32 elements.

For the first cipher tile, each pixel value in the initial plain tile is exclusive-or with the chaotic sequence *X* and a randomly selected integer value from hkey1. The pixels in a grayscale image have a single intensity value, whereas pixels in a color image are composed of three values representing the red, green, and blue channels (RGB). When performing operations on a single pixel in a color image, the operations are applied separately to each of the RGB channels. Notably, the encryption process for odd tiles in the plain image differs. A temporary tile is created by performing exclusive-or operations on odd tiles and the previously encrypted tile. These temporary tiles are further shuffled using *YC* and *YR* to obtain shuffled temporary tiles. The shuffled temporary tiles are then exclusive-or with the chaotic sequence X and a randomly selected integer value from hkey2. The value of hkey2 is generated by applying SHA256 to the concatenation of the previously encrypted tile and hkey1. Even tiles in the original image are encrypted by performing exclusive-or operations on the even tiles, previously encrypted tiles, chaotic sequence X, and a randomly selected integer value from hkey3. hkey3 is derived by applying SHA256 to concatenate the previously encrypted tile and the chaotic sequence X.

All the encrypted tiles are combined to produce the final single cipher image.

**Algorithm 11** *EncryptImage(img*, *D*, *R*_*t*_, *C*_*t*_, *x*, *r)*: This algorithm converts the original image into cipher image
**Input:** *img is the original image*, *D is the total tiles*, *R*_*t*_ *is the rows of tile*, *C\*_*t*_ *is the columns of tile*, *and (x*, *r) are the initial parameters of chaotic map*

**Output:** *cimg* is cipher image
*hkey1 = GetHashValue(img*,*””)*
*tiles←splitImage(img*, *D)*
*s_tiles* ← *ShuffleTiles(tiles*, *D*, *x*, *r)*
*X← keyGenerator(x*, *r*, *R*_*t*_*)*
*YC← indexGenerator(x*, *r*, *C*_*t*_*)*
*YR← indexGenerator(x*, *r+0*.*01*, *R*_*t*_*)*
*k← 1*
    for i ← *0 to R*_*t*_
       for *j* ← *0 to C*_*t*_
           *rand*, *x ← GenRanNum1* (*x*, *r*)
           $e\_tiles{\left[0\right]}_{i,j}\leftarrow tiles{\left[0\right]}_{i,j}⊕X_{k}⊕hkey1_{rand}$
           k←k+1
       endfor
    endfor
    for *ro ← 0 to D*
       if *ro%2 = 1*, then
      *temp_tile←empty tile of size R*_*t*_ *× C*_*t*_
        for *i← 0 to R*_*t*_
          for *j← 0 to C*_*t*_
            $temp\_tile_{i,j}\leftarrow \;tiles\;{\left[\;ro\;\right]}_{i,j}⊕\;e\_tiles\;{\left[ro-1\right]}_{i,j}$
          endfor
        endfor
      *shuffle_temp_tile← ShuffleTilePixels(temp_tile*,*YC*,*YR*, *R*_*t*_, *C*_*t*_*)*
      *hkey2← GetHashValue(tiles[ro-1]*,*hkey1)*
      *k← 0*
        for *i ← 0 to R*_*t*_
           for *j ← 0 to C*_*t*_
               *rand*, *x← GenRanNum2(x*,*r)*
               *$\;temp\_tile_{i,j}\leftarrow \;shuffle\_temp\_tile_{i,j}⊕X_{k}⊕\;hkey2_{rand}$*
               *k←k+1*
           endfor
         endfor
      *e_tile[ro]← temp_tile*
    else if *ro%2* = 0, then
      *k ← 0*
      *hkey3← GetHashValue* (e_tiles[ro-1]), X)
       for *i ← 0 to R*_*t*_
         for *j ← 0 to C*_*t*_
           *rand*, *x← GenRanNum2 (x*,*r)*
           *$temp\_tile_{i,j}\leftarrow \;tiles{\left[ro\right]}_{i,j}⊕e_{-}tiles{\left[ro-1\right]}_{i,j}⊕X_{k}⊕hkey3_{rand}$*
           *k←k+1*
        endfor
       endfor
      *e_tiles[ro]← temp_tile*
    endif
    endfor
*c_img← CombineImage(etiles*, *D)*
return *c_img*

The decryption algorithm accepts a cipher image as its input and transforms it into a plain image. During the conversion process, the decryption algorithm requires the cipher image, hkey1 value, the value of *D*, and the initial condition *(x*, *r)* for the chaotic sequence. The remaining secret values of *X*, *YR*, and *YC* are generated using the method detailed in the encryption algorithm. The encrypted image is partitioned into D = V×V encrypted tiles. The initial encrypted tile undergoes a transformation into a plain tile through an exclusive_or operation involving the first encrypted tile, a generated key sequence X, and hkey1. For the decryption of odd tiles, a temporary tile is formed by exclusively ORing the current odd tile with the previously decrypted tile. Subsequently, an inverse shuffling operation is applied to the temporary tile using chaotic sequences YC and YR to counteract the effects of shuffling during encryption. Following this, the shuffled temporary tile is exclusively ORed with chaotic sequence X and a randomly generated hkey2 to obtain the plain tile. Conversely, even tiles of the encrypted image are transformed into plain tiles by performing an exclusive_or operation on the current encrypted tile, the previously encrypted tile, chaotic sequence X, and a randomly generated hkey3. Finally, all decrypted tiles are reshuffled and combined to reconstruct the original file. The decryption process inverses the encryption process and is explained in Algorithm 12.

**Algorithm 12** *DecryptImage(c_img*, *hkey1*, *D*, *R*_*t*_, *C*_*t*_, *x*, *r)*: This algorithm coverts the cipher image into original image
**Input:** c_*img is the cipher image*, *hkey1 is the hash value of original*, *D is the total tiles*, *R*_*t*_ *is the total rows of tile*, *C*_*t*_ *is the total columns of tile*, *and (x*, *r) are the initial parameters of chaotic map*
**Output:** *img* is plain image
*e_tiles← SplitImage(c_img*, *D)*
*X← keyGenerator(x*, *r*, *R*_*t*_*)*
*YC← indexGenerator(x*, *r*, *C*_*t*_*)*
*YR← indexGenerator(x*, *r+0*.*01*, *R*_*t*_*)*
*k← 1*
    for i ← *0 to R*_*t*_
     for *j* ← *0 to C*_*t*_
       *rand*, *x ← GenRanNum1* (*x*, *r*)
       $tiles{\left[0\right]}_{i,j}\leftarrow e\_tiles{\left[0\right]}_{i,j}⊕X_{k}⊕hkey1_{rand}$
       k←k+1
     endfor
    endfor
    for *ro ← 1 to D*
     if *ro%2 = 1*, then
       *temp_tile←empty tile of size R*_*t*_ *× C*_*t*_
        for *i← 0 to R*_*t*_
    for *j← 0 to C*_*t*_
           $\;temp\_tile_{i,j}\leftarrow \;\text{tiles}\;{\left[\text{ro}\right]}_{i,j}⊕\;\text{e\_tiles}\;{\left[\text{ro}-1\right]}_{i,j}$
        endfor
       endfor
       *hkey2 ← GetHashValue(tiles[ro-1]*,*hkey1)*
       *k← 0*
      for *i ← 0 to R*_*t*_
       for *j ← 0 to C*_*t*_
         *rand*,*x ← GenRanNum2(x*,*r)*
         *$temp\_tile_{i,j}\leftarrow e\_tiles{\left[ro\right]}_{i,j}⊕X_{k}⊕hkey2_{rand}$*
         *k←k+1*
       endfor
    endfor
      *temp_tile← InverseShuffleTilePixels(temp_tiles*, *YC*, *YR*, *R*_*t*_, *C*_*t*_*)*
    for *i← 0 to R*_*t*_
     for *j ← to C*_*t*_
      *$temp\_tile_{i,j}\leftarrow \;temp\_tile_{i,j}⊕e_{-}tiles{\left[ro-1\right]}_{i,j}$*
     *endfor*
    *endfor*
    *tiles[ro] ← temp_tile*
   else if *ro%2 = 0*, then
    *k ← 0*
    *hkey3← GetHashValue* (*e_tiles[ro-1])*, *X*)
    for *i ← 0 to R*_*t*_
     for *j ← 0 to C*_*t*_
      *rand*,*x← GenRanNum2 (x*,*r)*
*
$temp\_tile_{i,j}\leftarrow e_{-}tiles{\left[ro\right]}_{i,j}⊕e_{-}tiles{\left[ro-1\right]}_{i,j}⊕X_{k}⊕\;hkey3_{rand}$
*
      *k←k+1*
     endfor
    endfor
    *tiles[ro]← temp_tile*
   *endif*
  *endfor*
*img = combineImage(tile*, *D)*
return *img*

## 4. Experimental setup

We used Python to implement all the image encryption schemes and evaluated them on the same system having specification Intel(R) Core (TM) i7-10870H CPU @ 2.20 GHz 2.21 GHz with 16GB RAM. We have used 8 RGB images of different sizes from CVG-UGR (https://ccia.ugr.es/cvg/dbimagenes/) and USC-SIPI (https://sipi.usc.edu/database/) image databases. The details of the images used in the experiment are shown in Table 2.

**Table 2:** Image dataset used for experiments.

No.	Name	M × N	Size	Bit Depth
1.	Jelly Beans	256 × 256	256 KB	24 bits
2.	Baboon	512 × 512	768KB	24 bits
3.	Peppers	512 × 512	768KB	24 bits
4.	Airplane(F-16)	512 × 512	768KB	24 bits
5.	House	512 × 512	768KB	24 bits
6.	Airport	1024 × 1024	3MB	24 bits
7.	Oakland	1024 × 1024	3MB	24 bits
8.	Car	1600 ×1067	4.88MB	24 bits

**Fig 8:**
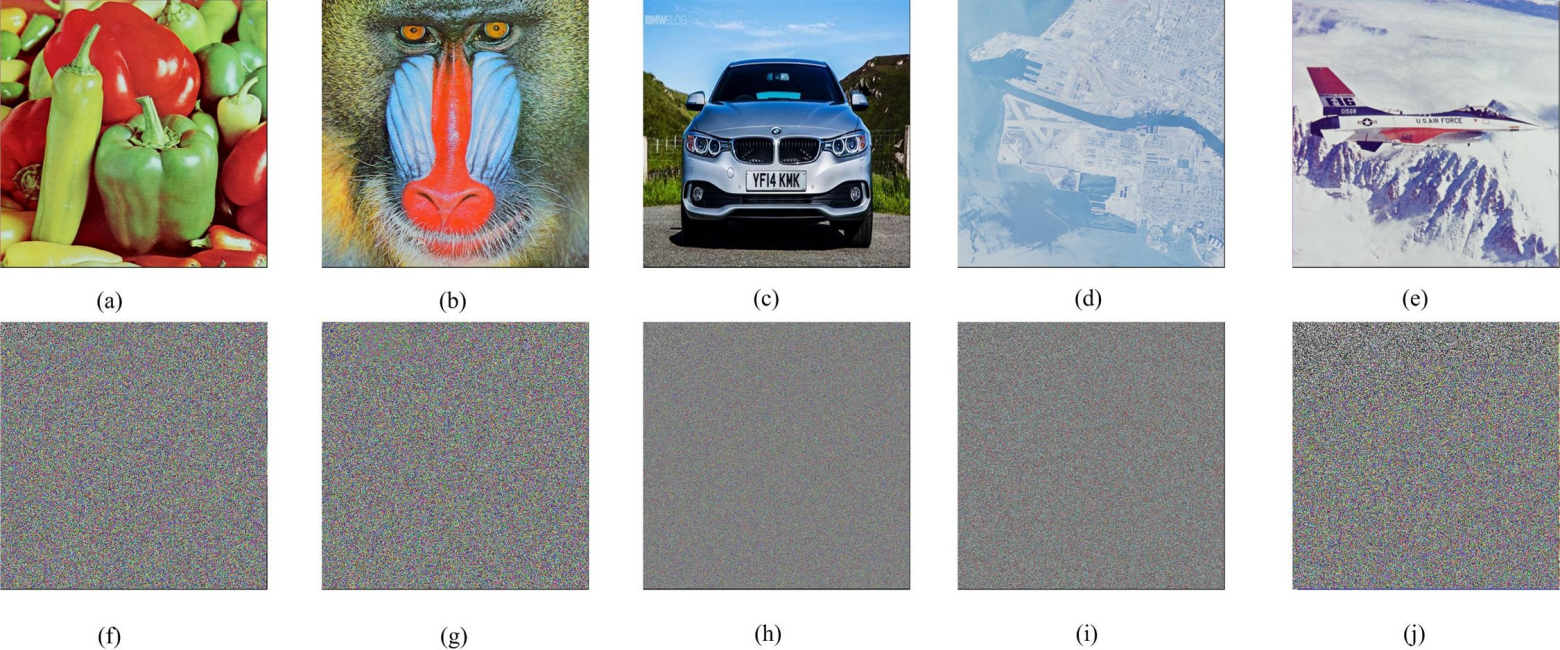
The plain images and their encrypted images using the proposed technique with tile size seven by seven.

We have used the JSMP map to generate a chaotic sequence because of its large chaotic regime—the value of *r∈[0*.*502*,*2000]*. The initial condition used for the chaotic map is *x*_*o*_ *= 0*.*4544* and *r = 3*.*91122*. The schemes are evaluated based on encryption/decryption time, entropy, Mean Square Error (MSE), correlation analysis, sensitivity analysis, and Peak Signal to noise ratio. The encryption results of various test images with tile sizes seven by seven are shown in Fig 8.

### 4.1 Encryption/decryption time

**Fig 9:**
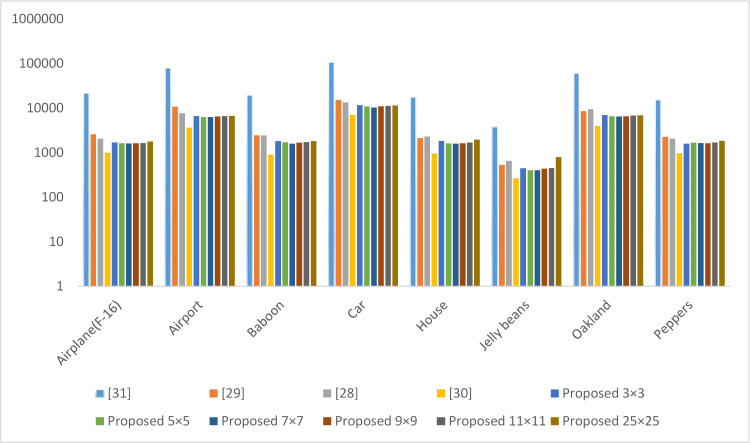
Encryption time in milliseconds.

**Fig 10:**
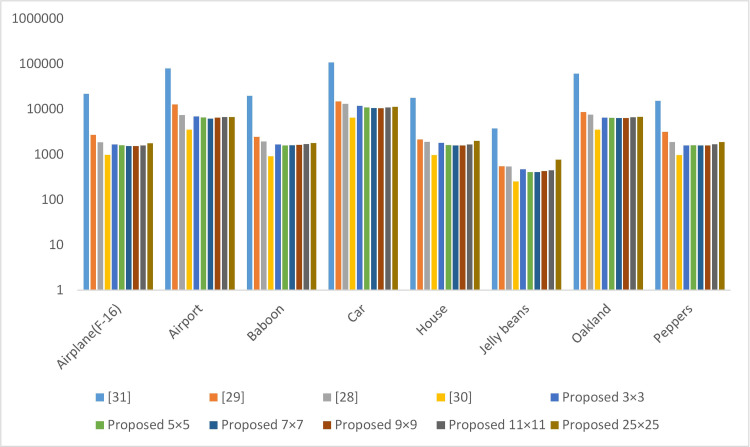
Decryption time in milliseconds.

This is the time taken by the encryption and decryption process in milliseconds. This time also depends on the system load at the time of evaluation. To overcome this issue, we have taken reading multiple times, and the average result is presented in Figs 9 and 10. The technique presented in [36] takes less time than others in encrypting and decrypting the image. The main reason is that the operations involved in [36] are lightweight. However, this scheme reveals the pattern in the encrypted image. Hence, it’s not secure to use. The scheme presented in [37] takes more time than others because of the operations, such as chaotic sequence generation, pixel transformation, shuffling of the pixels, exclusive-or, binary addition, and SBox operation.

The performance of the proposed scheme is better than the existing schemes [34, 35, 37] because the proposed scheme splits the image into multiple tiles of the same size. The size of the chaotic sequence generated by the proposed scheme is equal to the size of the pixels of a tile. The existing scheme [34, 35, 37] generates a chaotic sequence equal to the size of pixels in the plain image. The pixel in a tile is much less than the pixels in the original image. This is the main reason our scheme performs better than the existing one. As there are multiple readings of the proposed scheme with different tile distributions, The optimal performance of the proposed scheme is observed when we divide the image at seven by seven.

The decryption times show the same behaviours exhibited in the encryption process.

### 4.2 Histogram analysis

**Fig 11:**
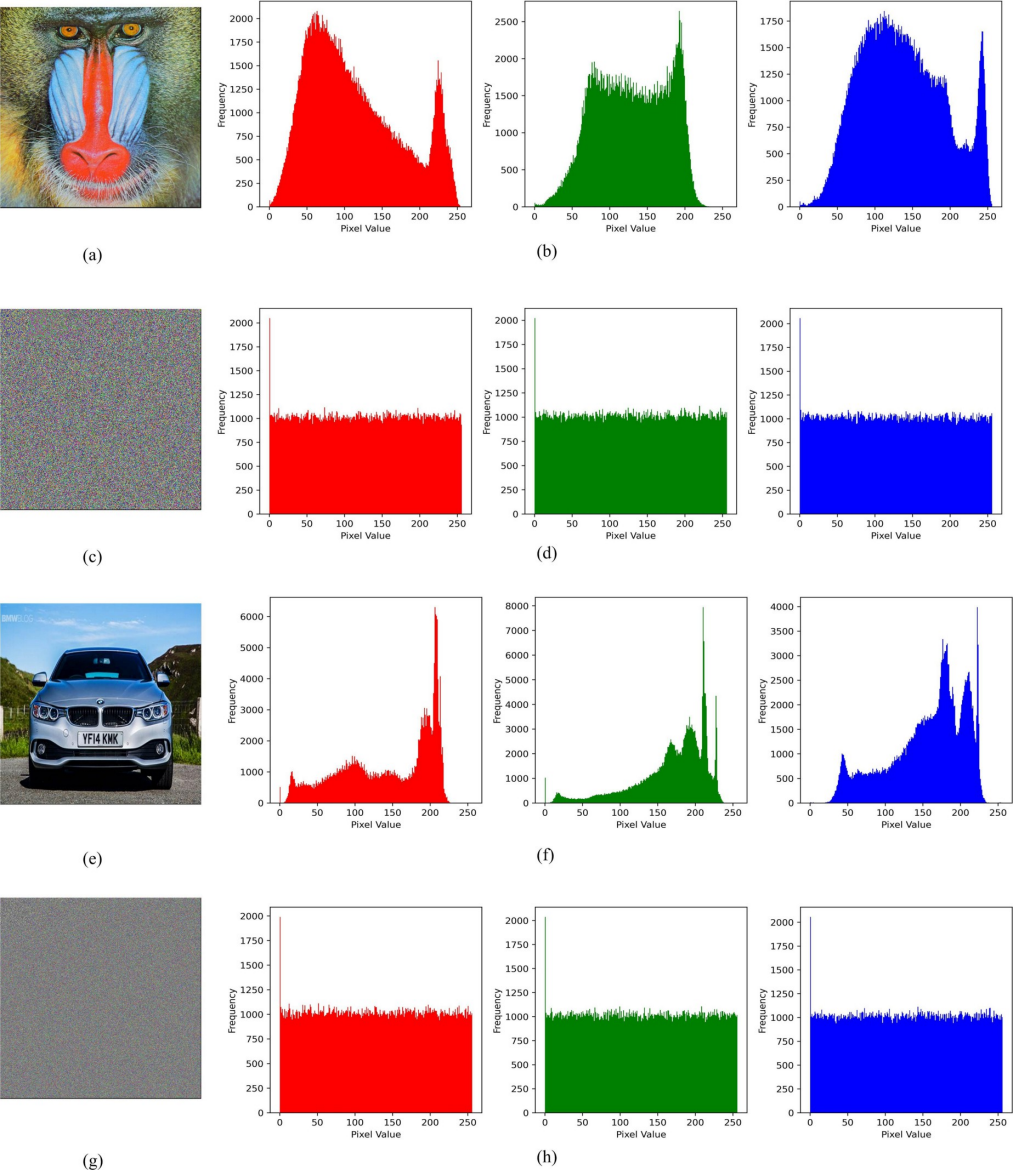
Analysis of histograms for two plaintext images encrypted using a proposed image encryption technique: (a) Original baboon image, (b) Histogram of the original baboon image, (c) Encrypted baboon image, (d) Histogram of the encrypted baboon image, (e) Original car image, (f) Histogram of the original car image, (g) Encrypted car image, and (h) Histogram of the encrypted car image.

The analysis of histograms offers valuable insights into the distribution patterns of pixel intensities inside images. A significant image generally exhibits a non-uniform distribution of pixel intensities throughout its entirety, whereas an encrypted image displays a uniform distribution. A good image encryption technique achieves a uniform distribution of pixels throughout the image. Fig 11 depicts the histogram of two images, namely the baboon and car, alongside their respective encrypted counterparts, which have been processed using the proposed image encryption technique. The analysis reveals that the distribution of images exhibits non-uniform characteristics, but the distribution of the encrypted image demonstrates uniform or flat attributes. Therefore, the technique under consideration aims to achieve a uniform distribution of RGB pixels throughout the image.

### 4.3 Correlation analysis

The correlation coefficient quantifies the degree of association between neighbouring pixels in an image. A higher correlation coefficient value indicates a significant association between the adjacent pixels of an image. If the correlation coefficient value is lower for an image, it indicates a weak or non-existent association between the neighbouring pixels of the image. If the image encryption technique fails to disrupt the correlation existing between neighbouring pixels within the encrypted image, an adversary may exploit this vulnerability through the utilization of a correlation attack, thereby obtaining valuable information. The calculation of the correlation coefficient value is determined by employing the subsequent formula:

$$e_{x}=\frac{\sum_{r=1}^{N}p_{i}}{N},\;\text{and}\;e_{y}=\frac{\sum_{r=0}^{N}p_{i+1}}{N}$$ (1)

$$d_{x}=\frac{\sum_{r=1}^{N}\left({\left(p_{i}-e_{x}\right)}^{2}\right)}{N},\;\text{and}\;d_{y}=\frac{\sum_{r=1}^{N}\left({\left(p_{i+1}-e_{y}\right)}^{2}\right)}{N}$$ (2)

$$con_{x_{i},x_{i+1}}=\frac{\sum_{i=1}^{N}\left(x_{i}-e_{x}\right)\left(x_{i+1}-e_{y}\right)}{N}$$ (3)

$$cor=\frac{con_{x_{i},x_{i+1}}}{d_{x}\times d_{y}}$$ (4)

**Fig 12:**
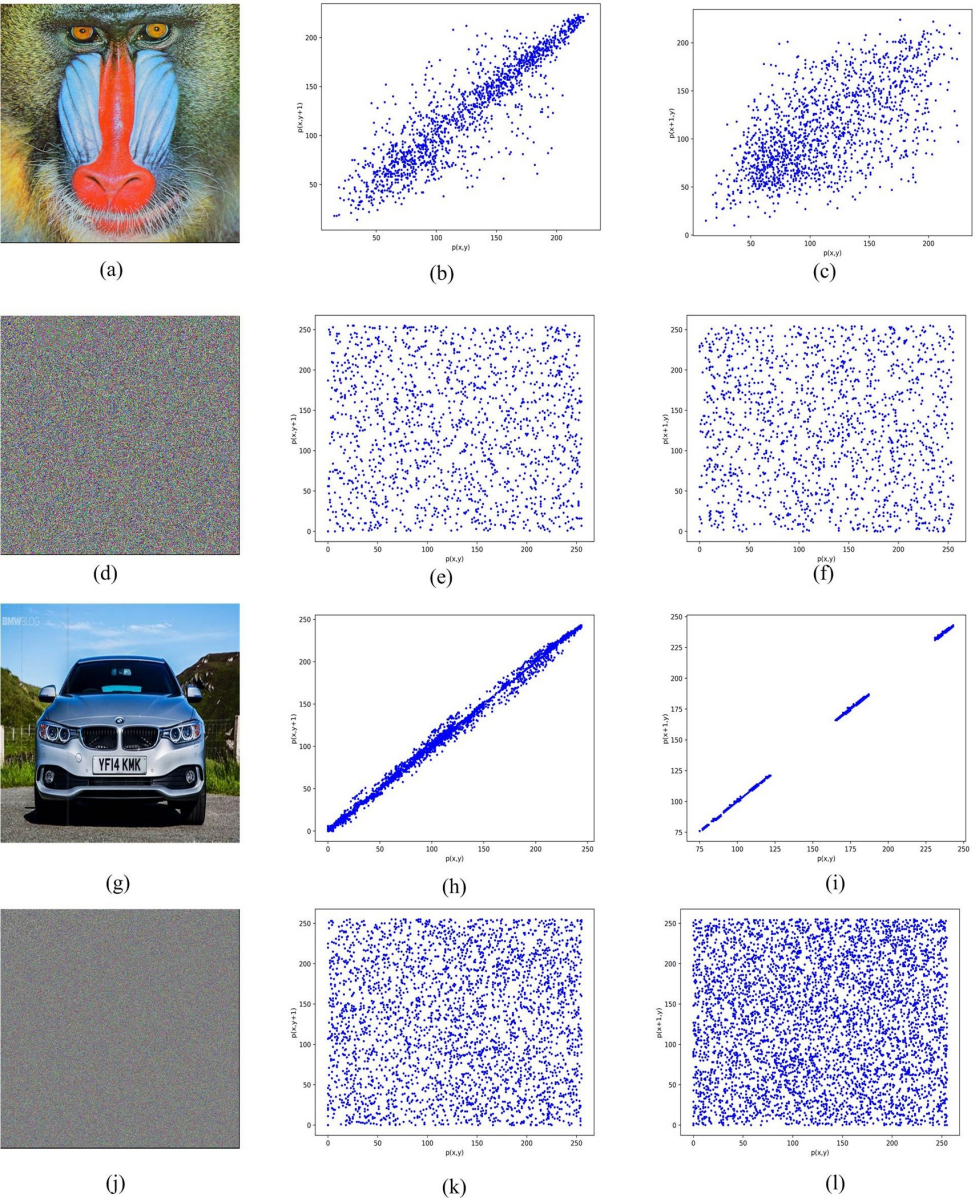
The relationship between adjacent rows and adjacent columns of plain image and encrypted image (a) plain image of baboon, (b) relationship between two adjacent columns of plain image baboon, (c) relationship between two adjacent rows of plain image baboon (d) encrypted image of baboon, (e) relationship between two adjacent columns of encrypted image baboon, (f) relationship between two adjacent rows of encrypted image baboon, (g) plain image of car, (h) relationship between two adjacent columns of plain image car, (i) relationship between two adjacent rows of plain image car (j) encrypted image of car, (k) relationship between two adjacent columns of encrypted image car, (l) relationship between two adjacent rows of encrypted image car.

The formulas are utilized to calculate the correlation between consecutive columns of an image. In this context, "*p*_*i*_" denotes the *i*^*th*^ pixel column, "*p*_*i+1*_" denotes the *(i+1)*^*th*^ pixel column, and "*N*" indicates the total number of pixels in a column. Fig 12 depicts the correlation between two consecutive columns and adjacent rows before and after the encryption process of two images utilizing the proposed image encryption technique. The results indicate that the suggested image encryption technique disrupts the correlation between horizontally and vertically neighbouring pixels within an encrypted image.

The proposed scheme is also compared with existing image encryption schemes. The obtained results are shown in Table 3.

**Table 3:** Correlation coefficient comparison.

	Plain Image	[37]	[35]	[34]	[36]	Proposed 3×3	Proposed 5×5	Proposed 7×7	Proposed 9×9	Proposed 11×11	Proposed 25×25
**Airplane(F-16)**	0.9984	0.7487	0.7185	0.7524	0.7493	0.7460	0.7477	0.7453	0.7354	0.7498	0.7520
**Airport**	0.9863	0.7478	0.7795	0.7484	0.7473	0.7478	0.7451	0.7467	0.7432	0.7479	0.7303
**Baboon**	0.9863	0.7488	0.7495	0.7491	0.7490	0.7488	0.7477	0.7448	0.7367	0.7384	0.7490
**Car**	0.9984	0.7480	0.7499	0.7495	0.7468	0.7472	0.7487	0.7463	0.7450	0.7458	0.7480
**House**	0.9961	0.7486	0.7333	0.7516	0.7485	0.7481	0.7473	0.7468	0.7457	0.7466	0.7509
**Jellybeans**	0.9987	0.7479	0.7205	0.7548	0.7482	0.7497	0.7472	0.7490	0.7547	0.7382	0.7574
**Oakland**	0.9980	0.7486	0.7237	0.7502	0.7487	0.7475	0.7453	0.7466	0.7425	0.7475	0.7312
**Peppers**	0.9969	0.7496	0.7269	0.7487	0.7482	0.7478	0.7460	0.7481	0.7350	0.7492	0.7500
**Average**	**0.9949**	**0.7485**	**0.7377**	**0.7506**	**0.7483**	**0.7478**	**0.7469**	**0.7467**	**0.7423**	**0.7454**	**0.7461**

The result shows a minor difference in each scheme’s average correlation coefficient value. The value of the correlation coefficient of [35] is better than our proposed scheme, with a minor difference. Alternatively, the correlation coefficient value of the proposed technique is better than [34, 36, 37]. The result also shows that our proposed scheme performs best with the tile size nine by nine. However, the performance of the proposed scheme is also acceptable with the tile size seven by seven.

### 4.4 Entropy analysis

Entropy measures the randomness or uncertainty of an image. A higher entropy value shows more randomness, making it difficult to get the information source. The calculated entropy value of the proposed technique and comparison with existing techniques is presented in Table 4.

**Table 4:** Entropy analysis.

	Original Image	[37]	[35]	[34]	[36]	Proposed 3×3	Proposed 5×5	Proposed 7×7	Proposed 9×9	Proposed 11×11	Proposed 25×25
**Airplane(F-16)**	13.956	17.926	16.819	17.870	17.069	17.777	17.887	17.881	17.529	17.659	17.306
**Airport**	6.830	8.000	7.987	7.153	8.000	7.999	7.993	7.998	7.981	7.999	7.881
**Baboon**	17.698	17.927	17.907	17.924	17.909	17.897	17.842	17.889	17.528	17.637	17.306
**Car**	14.427	20.268	18.291	20.195	18.677	20.133	20.238	20.194	20.147	20.229	20.046
**House**	16.301	17.925	17.712	17.917	17.789	17.870	17.844	17.890	17.530	17.638	17.305
**Jellybeans**	13.183	15.973	15.687	15.975	15.765	15.958	15.944	15.975	15.962	15.735	15.944
**Oakland**	15.261	19.722	19.079	19.633	18.989	19.627	19.605	19.664	19.528	19.691	19.020
**Peppers**	17.022	17.927	17.819	17.920	17.857	17.835	17.842	17.906	17.528	17.637	17.305
**Average**	**14.335**	**16.959**	**16.413**	**16.823**	**16.507**	**16.887**	**16.899**	**16.924**	**16.717**	**16.778**	**16.514**

The result shows that all the schemes introduce some randomness in encrypted images. The entropy of the proposed scheme with tile seven by seven is almost equal to [37] and is better than [34–36].

### 4.5 Mean Square Error (MSE) analysis

Mean Square Error (MSE) measures the difference between plain and encrypted images. The MSE value is calculated using the following formula:

$$\;the\;MSE=\left(\sum_{i=1}^{M}\sum_{j=1}^{N}{\left(P_{i,j}-E_{i,j}\right)}^{2}\right)/(M\times N)$$ (5)

Where *P* represents the plain image, *E* represents the encrypted image, and *M* and *N* are the image dimensions. The cryptosystem is considered strong if the MSE difference is high between the original and encrypted images. The MSE value of the proposed scheme and the existing scheme is presented in Table 5.

**Table 5:** MSE analysis.

	[37]	[35]	[34]	[36]	Proposed 3×3	Proposed 5×5	Proposed 7×7	Proposed 9×9	Proposed 11×11	Proposed 25×25
**Airplane(F-16)**	9937	10784	9846	9915	10078	9974	10029	10744	10509	12028
**Airport**	8677	8651	8691	8627	8687	8669	8704	8670	8698	8647
**Baboon**	8646	8610	8630	8582	8612	8660	8633	8911	8814	8269
**Car**	11110	10861	11112	11041	11136	11125	11149	11161	11127	11543
**House**	8777	9106	8788	8749	8799	8858	8837	9524	9263	10486
**Jellybeans**	9219	10021	9144	9118	9159	9208	9210	9022	9809	9665
**Oakland**	7398	7848	7398	7351	7436	7560	7488	7683	7444	10060
**Peppers**	7965	8424	8015	7958	8148	8157	8033	8673	8476	11768
**Average**	**8966.125**	**9288.125**	**8953**	**8917.625**	**9006.875**	**9026.375**	**9010.375**	**9298.5**	**9267.5**	**10308.2**

The result shows that the MSE value of the proposed technique increases with the tile size. The MSE value of the proposed scheme with tile size nine by nine and greater produces better results than existing schemes.

### 4.6 Sensitivity analysis

The secure image encryption schemes must support the avalanche effect. The cipher should be changed completely if there is a minor change in the plain image. The sensitivity analysis evaluates the Number of Changing Pixel Rate (NPRC) and Unified Averaged Changed Intensity (UACI). The ideal values of NPRC are greater than 99, and UACI is greater than 33 for a secure image encryption scheme. NPRC and UACI values are computed using the following formulas:

$$UACI=\sum_{i=1}^{N}\sum_{j=2}^{M}\frac{\left|C1_{i,j}-C2_{i,j}\right|}{255\times M\times N}\times 100$$ (6)

To evaluate the value of UACI, two identical images having only a one-pixel difference are encrypted, and the cipher images are represented with C1 and C2.


$$M_{i,j}=\left\{\begin{array}{l} 1,\;if\;C1_{i,j}\neq C2_{i,j}\\ 0,\;if\;C1_{i,j}\neq C2_{i,j} \end{array}\right.$$ (7)

$$NPRC=\frac{1}{N\times M}\left(\sum_{i=1}^{N}\sum_{j=1}^{M}M_{i,j}\right)\times 100$$ (8)

While testing this scheme in our experimental setup, the key’s value remains the same for both encrypted images with a one-pixel difference. The obtained results are presented in Tables 6 and 7.

**Table 6:** UACI analysis.

	[37]	[35]	[34]	[36]	Proposed 3×3	Proposed 5×5	Proposed 7×7	Proposed 9×9	Proposed 11×11	Proposed 25×25
**Airplane(F-16)**	33.45	0.00	3.59	0.00	33.53	33.31	33.49	33.99	32.78	34.03
**Airport**	33.43	0.00	3.25	0.00	33.16	33.09	33.32	32.93	33.35	31.90
**Baboon**	33.41	0.00	3.16	0.00	33.76	33.29	33.45	33.38	32.76	31.95
**Car**	33.49	0.00	2.94	0.00	33.98	33.69	33.41	33.22	33.42	32.92
**House**	33.45	0.00	2.99	0.00	33.46	33.14	33.45	32.53	32.70	31.97
**Jellybeans**	33.51	0.00	2.53	0.00	33.81	33.52	33.60	32.73	34.41	34.46
**Oakland**	33.46	0.00	2.14	0.00	34.26	33.49	33.58	33.12	33.47	31.89
**Peppers**	33.46	0.00	2.10	0.00	33.88	33.70	33.56	32.54	32.74	31.85
**Average**	**33.46**	**0.00**	**2.84**	**0.00**	**33.73**	**33.40**	**33.48**	**33.06**	**33.20**	**32.62**

**Table 7:** NPRC analysis.

	[37]	[35]	[34]	[36]	Proposed 3×3	Proposed 5×5	Proposed 7×7	Proposed 9×9	Proposed 11×11	Proposed 25×25
**Airplane(F-16)**	100.00	0.00	95.88	0.00	99.60	99.25	99.66	99.57	97.44	98.15
**Airport**	99.61	0.00	95.33	0.00	99.49	98.86	99.24	98.26	99.41	95.00
**Baboon**	100.00	0.00	95.32	0.00	99.69	98.83	99.25	99.64	97.31	95.02
**Car**	100.00	0.00	94.93	0.00	99.38	99.42	99.09	98.72	99.29	98.02
**House**	100.00	0.00	95.07	0.00	99.65	98.87	99.23	96.51	97.40	95.00
**Jellybeans**	100.00	0.00	89.29	0.00	99.67	99.31	99.61	99.65	99.63	99.65
**Oakland**	100.00	0.00	89.60	0.00	99.42	98.85	99.24	98.27	99.43	94.99
**Peppers**	100.00	0.00	88.82	0.00	99.56	99.57	99.63	96.55	97.30	95.00
**Average**	**99.95**	**0.00**	**93.03**	**0.00**	**99.56**	**99.12**	**99.36**	**98.40**	**98.40**	**96.35**

It is observed from the result that the proposed scheme is secure against differential attack as the value of both parameters is in the acceptable range in most of the cases. The result of the schemes presented in [35, 36] is nearly equal to zero, and the result of [34] is also not in an acceptable range. The only existing scheme that protects against differential attack is [37]. The performance of our proposed technique is better than [37], with a tile size seven by seven.

### 4.7. NIST 800–22 test suits

We have tested our proposed image encryption scheme using NIST 800–22 test suits to validate the randomness in the encrypted image of a baboon of size 512 by 512. For each NIST 800–22 test, a P-value exceeding 0.01 is required for successful passage. The outcomes presented in Table *8* affirm that the encrypted image successfully passed all the randomness tests outlined in NIST 800–22.

**Table 8:** The validation of the proposed encryption scheme based on NIST 800–22 test suits.

Statistical Test	P-Value	Proportion	Status
Frequency	0.038879	10/10	Successful
BlockFrequency	0.122325	10/10	Successful
CumulativeSums	0.739918	10/10	Successful
Runs	0.911413	10/10	Successful
LongestRun	0.534146	10/10	Successful
Rank	0.739918	10/10	Successful
FFT	0.534146	10/10	Successful
NonOverlappingTemplate	0.739918	10/10	Successful
OverlappingTemplate	0.534146	10/10	Successful
Universal	0.616754	10/10	Successful
ApproximateEntropy	0.284345	8/10	Successful
RandomExcursions	0.347644	3/3	Successful
RandomExcursionsVariant	0.325360	3/3	Successful
Serial	0.269952	2/3	Successful
LinearComplexity	0.991468	10/10	Successful

**Note:** Each statistical test requires a minimum pass rate of approximately 8, except for the random excursion (variant) test, which has a minimum pass rate of approximately 2. These criteria apply to binary sequences with a sample size of 10 for most tests and a sample size of 3 for the random excursion (variant) test.

### 4.8. Contrast analysis

This analysis provides information on the difference in intensity of the darkest and brightest parts of an image. A greater contrast value indicates a more significant difference, while a lower value signifies a more even distribution of tones. When the image encryption algorithm relies on pixel values or frequency components, a cryptic image with an elevated contrast value becomes challenging for an attacker to decipher. Table 9 presents the contrast value of the original image and encrypted image using the proposed image technique. The results confirm that the proposed image encryption substantially enhances the contrast level in the encrypted image.

**Table 9:** Contrast analysis of the proposed image encryption scheme.

	Plain image Contrast Value	Total tiles 3 × 3	Total tiles 5 × 5	Total tiles 7 × 7	Total tiles 11 × 11	Total tiles 25 × 25
Airplane(F-16)	0.2662	0.4983	0.4421	0.4394	0.4233	0.4336
Baboon	0.3292	0.4132	0.3972	0.3901	0.4336	0.4233
Oakland	0.1231	0.4640	0.4380	0.4369	0.4287	0.4785
House	0.2628	0.4465	0.3971	0.3982	0.4228	0.4556

### 4.9. Key space analysis

The encryption and decryption process involves three types of secret keys. The first key, with a size of 256 bits, represents the hash value of the original image. The second key comprises the initial parameters of the logistic map, essential for generating random numbers. Lastly, the third key consists of the initial parameters of the JSMP map, used to generate key sequences (X, Y, and Z) to impart confusion and diffusion properties to the encrypted image. The chaotic range of the logistic map spans from 3.57 to 4.0, defined by two initial parameters: x_0_ and r. The value of x_0_ falls within the range of 0 to 1, while the parameter r ranges from 3.57 to 4.0. Notably, x_0_ for the logistic map has 15 varied digits in its decimal fraction, and the parameter r has 16 decimal fraction digits. Similarly, the chaotic range of the JSMP map extends from 0.502 to 2000, governed by two initial parameters: x_0_ and r. The x_0_ parameter of the JSMP map consists of 15 varied digits in its decimal fraction, while the r parameter comprises 17 decimal fraction digits. Consequently, the total key space required for a brute-force attack can be determined using the following equation:

$$\;Key\;Space\;=2000\times 10^{15}\times 10^{17}\times 10^{15}\times 10^{16}\times 2^{256}$$ (9)

Therefore, the analysis above validates the security of the suggested image encryption technique against brute-force attacks. To enhance the key space, an option is to incorporate a hash function that generates a hash on the original image exceeding 256 bits, such as SHA-512, SHA-3, and so forth. Within our proposed image encryption scheme, we opted for SHA-256 from the SHA family due to its security features and lower processing requirements compared to other SHA members. It’s worth noting that in the future, should heightened security be necessary, there is flexibility to substitute SHA-256 with other members of the SHA family.

### 4.10. Security analysis

The proposed image encryption scheme undergoes evaluation through known plaintext attacks, known ciphertext-only attacks, chosen plaintext attacks, and chosen ciphertext attacks. In a known ciphertext-only attack, the attacker possesses one or more ciphertexts exclusively. To decipher this ciphertext, the attacker must possess knowledge of the original image to generate the initial secret key and two additional keys, serving as initial parameters for chaotic sequence generation. Since the encrypted image does not disclose any key-related information, executing a known ciphertext-only attack to unveil key secrets is infeasible. The ciphertext-only attack provides no insights into either the keys or the original image. Conversely, in a known plaintext attack, the attacker possesses information about both the encrypted image and its corresponding cipher image, aiming to extract key details. Here, the attacker has access to the original image, enabling the derivation of the initial 256-bit key through hashing the original image. However, obtaining the remaining two keys, associated with the logistic map and JSMP map’s initial parameters remains elusive even after acquiring knowledge of the first key. Consequently, the proposed system exhibits security against known plaintext attacks. In a chosen plaintext attack, the attacker possesses the capability to utilize the encryption oracle. Through this ability, the attacker encrypts chosen messages, analyzing the resulting ciphertext patterns to get secret information. However, even in this scenario, obtaining details about the encryption keys or the encrypted image proves elusive. This is because the initial key used in the image encryption process is derived from the hash value of the original image itself. Consequently, despite ciphertext observation, the attacker remains unable to extract any meaningful information. Similarly, in a chosen ciphertext attack, the attacker can employ the decryption oracle to decrypt messages of their choosing. Nevertheless, this capability does not facilitate the acquisition of secret information for the same reason outlined previously. Consequently, the proposed encryption technique provides protection against various attack scenarios, including known plaintext attacks, known ciphertext-only attacks, chosen plaintext attacks, and chosen ciphertext attacks.

### 4.11. Time complexity

The key operations within the proposed image encryption method encompass image splitting, tile shuffling, key generation, encryption, decryption, inverse splitting, and tile combination. The time complexity associated with both the image splitting and tile combination processes is O(T), where T signifies the total number of tiles. Likewise, the shuffle and reshuffle operations exhibit a time complexity of O(T). The encryption and decryption algorithms contribute a time complexity of O(D × D × T), where D denotes the number of rows or columns in a tile, and T represents the total number of tiles. The time complexity of the key generation module is O(D × D). Consequently, the comprehensive complexity of the proposed image encryption method is expressed as O(D × D × T). Table 10 illustrates a comparison of time complexities between the proposed image encryption technique and existing image encryption techniques. The analysis affirms that the time complexity of the proposed image encryption outperforms that of the existing image encryption schemes.

**Table 10:** Time complexities comparison.

	Time Complexity
Proposed Technique	O(D × D × T)
[37]	O(k×M×N)
[35]	O(8×M×N).
[34]	O(M × N)
[36]	O(M × N)

Note: M represents the total rows in the original image, N represents the total columns in the original image, k is constant, D denotes the number of rows or columns in a tile, and T represents the total number of tiles.

## 5. Conclusion

Image data is among the most important data being shared on the Internet. Many sensitive applications, such as Healthcare, Internet of Battlefield Things (IoBTs), Satellite, Surveillance applications, and many more, send image data over the Internet. The image data of such applications must be secured to avoid any physical or financial loss. The resource limitation of a few of the applications described above restricts the usage of traditional techniques for image encryption. Hence, there is a need for a lightweight and secure image encryption scheme. The object of this research study is to propose a lightweight and secure image encryption technique for resource-constrained environments. The result shows that the proposed image encryption scheme with the seven-by-seven tiles gives optimal results compared to the existing lightweight image encryption schemes. Moreover, the security analysis also indicates that the proposed scheme is secure against differential attacks. The brute force attack is also impossible because of the long chaotic regime of the JSMP map and the 256-bit key used as the encryption key.

The proposed image encryption scheme functions seamlessly with images that can be perfectly divided into equal-sized tiles. In cases where an image possesses dimensions that do not permit division into tiles of equal size, the algorithm refrains from encrypting less than 0.5% of the final rows and columns. Nonetheless, this impact remains imperceptible to the human eye. Our objective is to rectify and eliminate this limitation in future iterations.
